# Combined MitraClip and TriClip therapy in patients with severe mitral and tricuspid regurgitation: determinants and prediction of mid-term outcomes

**DOI:** 10.3389/fcvm.2026.1855406

**Published:** 2026-08-05

**Authors:** Giuseppe D´Ancona, Mohamed Al Saidi, Antonia Ourani, Jörg Hering, Robert Gramlich, Sebastian Feickert, Kristof Biernath, Plamen Kochev, Jonas Michael Bodanowitz, Hüseyin Ince

**Affiliations:** 1 Department of Cardiology, Vivantes Klinikum am Urban und Neukölln, Berlin, Germany; 2 Heart Center, Medical University Rostock, Rostock, Germany

**Keywords:** mitral, tricuspid, regurgitation, edge-to-edge, repair, percutaneous, combined, staged

## Abstract

**Background:**

Patients with combined severe mitral (MR) and tricuspid regurgitation (TR) constitute a high-risk cohort with advanced right-heart disease, reduced survival, and frequent heart failure hospitalizations. While transcatheter edge-to-edge repair (TEER) is guideline-endorsed for severe MR and increasingly adopted for TR, long-term evidence on combined dual-valve TEER remains limited. The TRIVALVE score has shown prognostic utility in transcatheter tricuspid interventions but has not been specifically evaluated in patients requiring combined TEER.

**Methods:**

We prospectively enrolled 54 consecutive patients with severe MR and TR undergoing combined TEER by means of MitraClip® and TriClip®. Baseline clinical, echocardiographic, and laboratory data were collected. The primary endpoint was all-cause mortality; the secondary endpoint was a composite endpoint of all-cause mortality or heart failure rehospitalization (cumulative events). Survival and cumulative events were estimated by Kaplan–Meier analysis, determinants were identified by logistic regression, and prediction models were built using random-forest, logistic regression, and gradient boosting.

**Results:**

Median TRIVALVE score was 2.0 (IQR 1.0–3.0) and median EuroSCORE II was 4.5 (IQR: 3.0–7.6). Thirty-day mortality was 5.5% (3/54). Median clinical follow-up was 607 days (IQR 289–906). Estimated 1-year and 2-year survival was 91.8% and 64.5%, with 74.2% and 52.7% freedom from mortality or heart failure rehospitalization. In the regression analysis, both TRIVALVE score (OR=2.19; 95% CI: 1.06–4.53; *p* = 0.034) and baseline level of hemoglobin (OR=0.64; 95% CI: 0.43–0.96; *p* = 0.029) were significantly associated with mortality. The composite of all-cause mortality or heart-failure rehospitalization was independently predicted by the TRIVALVE score (OR=2.53; 95% CI: 1.26–5.09; *p* = 0.009) with the component of pre-procedural atrial fibrillation and right heart failure having the strongest prediction. Random forest showed the highest discriminative performance for prediction of follow-up mortality (AUC 0.79; Sens. 0.84; Spec. 0.66; 95% CI 0.63–0.96), and the composite of all-cause mortality or heart-failure rehospitalization (AUC 0.86; Sens.0.8; Spec. 0.67; 95% CI 0.718–0.976).

**Conclusion:**

Combined dual-valve TEER achieved acceptable mid-term survival and event-free outcomes. Outcomes were driven more by baseline disease burden than by procedural success alone. Lower baseline hemoglobin and a higher TRIVALVE score were both linked to worse prognosis, with atrial fibrillation and right heart failure as its most influential components, highlighting the contribution of chronic atrial remodeling and congestion to recurrent events. These findings are hypothesis-generating and require external validation.

## Introduction

Patients with combined severe mitral regurgitation (MR) and tricuspid regurgitation (TR) represent a particularly high-risk population in whom chronic left-sided volume overload, pulmonary vascular remodeling, venous congestion, and progressive right-heart dysfunction frequently coexist. In these patients, TR is not merely an innocent bystander of left-sided disease and observational data have shown that functional TR is independently associated with excess mortality in heart failure populations. Consequently, concomitant moderate-or-greater TR identifies a subgroup with more advanced disease and worse outcomes among patients undergoing mitral transcatheter edge-to-edge repair (TEER) (1–4).

Over the past decade, TEER has transformed the therapeutic landscape for patients considered poor surgical candidates. In secondary MR, the COAPT trial established that mitral TEER, when added to guideline-directed medical therapy in carefully selected patients, reduces heart failure hospitalization and all-cause mortality, with sustained benefit at 5 years (5, 6). In severe symptomatic TR, transcatheter tricuspid TEER has evolved from feasibility studies and registry experience to randomized evidence: the TRILUMINATE Pivotal trial demonstrated that tricuspid TEER is safe, reduces TR severity, and improves health status, while subsequent analyses confirmed meaningful gains in symptoms, function, and quality of life (7, 8).

However, most of the available literature still comes from isolated-valve treatment paradigms. Patients with severe MR and severe TR treated percutaneously on both valves remain underrepresented in randomized studies, and the optimal sequencing, timing, and selection criteria for a dual-valve transcatheter strategy remain uncertain (9–12). Small observational cohorts and registry analyses suggest that combined or concomitant mitral-tricuspid TEER is feasible and may offer clinical advantages over isolated mitral repair in selected patients, but these data also indicate that outcomes are heavily conditioned by disease stage, particularly the extent of right-heart remodeling and right ventricular (RV) dysfunction at the time of intervention (9–12).

This issue makes risk stratification especially relevant. Recent work has highlighted the prognostic value of integrated right-heart disease burden in patients undergoing transcatheter tricuspid intervention. TRISCORE, originally developed for isolated tricuspid surgery, has subsequently been examined in transcatheter cohorts, while the newer TRIVALVE score was specifically designed to predict death or rehospitalization after transcatheter tricuspid valve intervention. Both tools move beyond valve anatomy alone and capture systemic disease severity through markers such as atrial fibrillation, renal-hepatic dysfunction, signs of right-heart failure, and ventricular impairment (13, 14).

Against this background, studying patients treated percutaneously for combined severe MR and TR is important for two reasons. First, this is a clinically relevant population in whom the decision to treat one valve, both valves, or to stage treatment remains poorly standardized. Second, in this setting, prognosis may be driven less by procedural feasibility alone and more by the burden of right-heart disease present at baseline.

The present study therefore sought to evaluate the long-term clinical and echocardiographic outcomes of combined, single-center, MitraClip® and TriClip® therapy in patients with combined severe MR and TR, and to identify predictors of follow-up mortality and of the composite endpoint of mortality or heart failure rehospitalization, with particular attention to the roles of TRIVALVE score, with its individual score components, and to simplified markers of overall patient´s frailty.

Because our cohort comprises combined MR/TR disease treated with a combined treatment strategy, we regard TRIVALVE score application in this context as an exploratory external use rather than a formal validation. The Selection of the TRIVALVE Score has practical reasons. First, it integrates parameters that reflect both mitral and tricuspid disease and the broader cardiac and end-organ burden these patients carry, including the interrelated systemic consequences of biventricular valve disease, features well suited to a combined MR/TR population. Moreover, the TRIVALVE score is simple enough to calculate at the bedside, with parameters that are systematically captured before the interventions which supports its use in routine clinical decision-making.

## Methods

### Study population

We prospectively enrolled 54 consecutive patients treated from 9/2014 to 2/2026 with combined severe MR and TR undergoing either simultaneous (during the same operative session) or staged combined TEER with MitraClip® followed by TriClip® at Vivantes Klinikum Urban/Neukölln in Berlin, Germany.

### Data collection

Because TR management followed MR management, for this specific study we have considered as baseline values those clinical, echocardiographic, and laboratory data available immediately before the hospital admission for TEER of MR.

Since the TRIVALVE score reflects tricuspid-specific anatomical and procedural risk, it was assessed immediately before TriClip®. This approach allowed baseline patient vulnerability to be captured before the treatment pathway, while the TRIVALVE score represented the patient's condition at the time of tricuspid intervention.

Baseline clinical, echocardiographic, and laboratory data were collected in our Institutional electronic chart prior to intervention. Echocardiographic studies were performed and analyzed in the institutional core echocardiography laboratory by members of the echocardiography team. At baseline, guideline-recommended parameters for grading atrioventricular valve regurgitation were obtained in all patients where feasible, including regurgitant volume, vena contracta, PISA, EROA, and systolic pulmonary artery pressure (sPAP). Mitral regurgitation was graded using the three-grade classification, and tricuspid regurgitation using the five-grade scheme, which corresponds to the extended tricuspid grading classification. Central venous pressure was estimated from echocardiographic criteria and assigned to one of three levels (3, 8, or 15 mmHg); where these data were missing, a default value of 8 mmHg was applied. After the intervention, follow-up assessment comprised left ventricular ejection fraction, mitral and tricuspid regurgitation severity, the transvalvular pressure gradients across both valves, and sPAP.

Follow-up data collected at discharge after the last intervention included heart failure rehospitalization recorded in our electronic chart. Admissions within the Vivantes hospital network, comprising more than nine hospitals, were captured systematically through shared electronic records. All surviving patients were followed-up with outpatient visits and trans-thoracic echocardiography, in our facility or in the offices of the referring cardiologists, at 30-days, 3-months and 6-months after each intervention. Moreover, to confirm the life status and additional hospitalizations, patients were telephonically contacted on 3/2026. The study has been submitted to and approved from the Rostock University Ethical Committee, and it is part of the doctoral thesis of one of the authors (M.A.).

### Study endpoints

The primary endpoint was all-cause follow-up mortality. The secondary endpoint was a composite of all-cause mortality or heart failure rehospitalization.

Procedural success was defined separately for each procedure. TEER of mitral valve success was defined as a reduction of MR by at least one grade to an absolute level of 2 + or lower. TEER of TR success was defined as a reduction of TR by at least one grade, based on the five-grade tricuspid classification. Overall procedural success for both interventions, whether simultaneous or staged, required a reduction of both mitral and tricuspid regurgitation by at least one grade on post-procedural echocardiography, together with no need for unplanned surgical intervention on the atrioventricular valves.

### Statistical analysis

Continuous variables are presented as mean ± standard deviation or median (interquartile range) as appropriate, and categorical variables as counts and percentages. Between-group comparisons were performed using Student's t-test or Mann–Whitney U test for continuous variables and *χ*^2^ or Fisher's exact test for categorical variables, as appropriate. Pre- and post-intervention variables were compared using paired t-test, Wilcoxon signed-rank test, and McNemaŕs test according to the type of data.

Time-to-event analyses were performed using Kaplan–Meier estimates. The primary endpoint was all-cause mortality, and the secondary endpoint was the composite of all-cause mortality or heart failure rehospitalization.

### Inferential analysis

In-hospital deaths were included in the endpoints to avoid exclusion of clinically relevant early events and to reduce potential survivor bias.

Four separate logistic regression models were constructed: (1) overall mortality, including both in-hospital and follow-up deaths; (2) cumulative adverse events, defined as composite of all-cause mortality and heart-failure rehospitalization; (3) overall mortality using selected individual components of the TRIVALVE score instead of the composite score to reduce potential collinearity; 4) cumulative adverse events using selected individual components of the TRIVALVE score instead of the composite score to reduce potential collinearity.

Given the limited number of events, particular care was taken to avoid model overfitting. Candidate variables were screened through univariate analyses, and only predictors demonstrating significant association with the outcome (*p* ≤ 0.05) were retained for multivariable modeling. The final logistic regression models were intentionally restricted to a small number of predictors to ensure model parsimony. Model calibration was assessed using the Hosmer–Lemeshow goodness-of-fit test. Results are reported as odds ratios (ORs) with 95% confidence intervals (CIs).

All statistical analyses were performed using IBM SPSS Statistics (version 28). A two-sided *p*-value <0.05 was considered statistically significant.

### Exploratory predictive model

To complement inferential analysis, exploratory and hypothesis generating predictive modeling was performed using supervised machine learning techniques implemented in Orange Data Mining software (version 3.40.0). Variables identified as independent predictors of mortality and cumulative events (mortality and rehospitalization) in logistic regression were used as input features. Three classification algorithms were evaluated:

1. logistic regression with ridge regularization,
2. random forest (10 trees), and
3. gradient boosting.

Model performance was evaluated using five-fold cross-validation. Missing predictor values were handled using mean imputation within the Orange preprocessing workflow. Continuous variables were standardized prior to model training. Preprocessing was incorporated into the Test & Score cross-validation procedure so that imputation and standardization were performed within the cross-validation framework, thereby minimizing information leakage between training and test folds. No synthetic oversampling or class-balancing procedures were applied. Although the outcome distribution was imbalanced, the degree of imbalance was considered acceptable for exploratory analyses. Discriminatory performance was assessed using the area under the receiver operating characteristic curve (AUC), reported together with approximate 95% confidence intervals.

## Results

### Descriptive statistics

A total of 54 were included in the study.

Two patients (3.7%) underwent M-TEER in 2014 and 2018, while all remaining 52 procedures (96.3%), including all T-TEER, were performed between 03.2019 and 03.2026.

For M-TEER, the fourth-generation clip was mainly used, with the NTW system implanted in the majority of cases (43 cases, 79.6%). In the earliest TR cases, before dedicated tricuspid devices were available, third-generation MitraClip® systems were used (XTR, *n* = 6). Later procedures used the first generation TriClip (XT, *n* = 4) and, again off-label, the fourth-generation MitraClip® (XTW, *n* = 8). The fourth generation TriClip® (XTW, *n* = 31) accounted for most cases, and from October 2025 the fifth generation TriClip® (XTW) was implanted.

Seven patients (13%) had simultaneous TEER of MR and TR, the remaining 47 (87%) had first MR followed by TR TEER at a median distance of 143.5 days (IQR: 63–224 days).

Median TRIVALVE score, calculated before TEER of TR was 2.0 (IQR 1.0–3.0) and median EuroSCORE II was 4.5 (IQR: 3.0–7.6). All patients had successful placement of at least one clip in the mitral position, and in one case (1.8%) TEER of TR was abandoned due to unsuitable anatomy.

A median of 2 clips (min 1-max 4) was required for MR TEER and a median of 2 clips (min 1- max 4) for TR TEER, for a total median value of 4 clips per patient (min 2- max 7).

**Figure 1:**
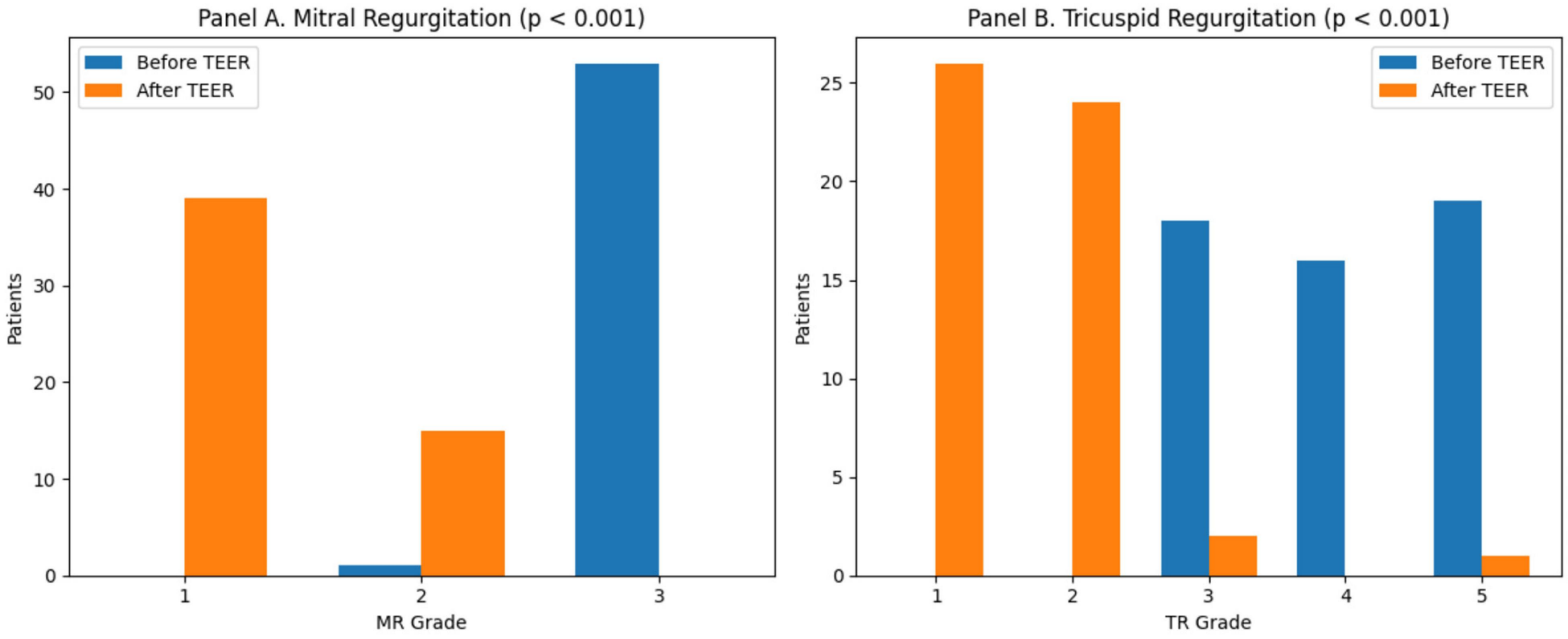
Change in MR and TR degree after TEER. **(A)** MR severity significantly improved after TEER, with 98.1% of patients showing reduction in MR grade (Wilcoxon signed-rank-test, *p* < 0.0001). **(B)** Similarly, TR showed a significant reduction, with 96.2% of patients improving (Wilcoxon signed-rank-test, *p* < 0.0001).

Figure 1 summarizes change in MR and TR degree after TEER. MR severity significantly improved after TEER, with 98.1% of patients showing reduction in MR grade (Wilcoxon signed-rank-test, *p* < 0.0001). Similarly, TR showed a significant reduction, with 96.2% of patients improving (Wilcoxon signed-rank-test, *p* < 0.0001). In one case (1.8%) TR could not be treated with TEER and remained torrential (grade 5). In one case (1.8%) TR passed from massive (grade 4) to severe (grade 3), and in one case (1.8%) TR remained unchanged (severe, grade 3).

NYHA functional class significantly improved after TEER of MR and TR intervention, with two-thirds of patients experiencing at least one-class improvement and no cases of deterioration. This was confirmed by paired non-parametric testing (Wilcoxon signed-rank test, *p* < 0.00001), indicating a robust functional benefit following dual-valve TEER.

In-hospital/thirty-day mortality was 5.5% (3/54): one patient died 10 days after TR-TEER having experienced pericardial tamponade and ischemic cerebral stroke, one patient died 5 days after failed TR TEER (unsuitable anatomy) as result of multi-organ failure, and one patient died for cardiac arrest in the cardiological ward 7 days after successful TR-TEER (no autopsy was granted and cause of death remains unclear).

Median hospitalization after TEER of MR was 4 days (IQR: 4–5 days) and after TEER of TR 4 days (IQR: 3–5 days).

### Survival analysis

**Figure 2:**
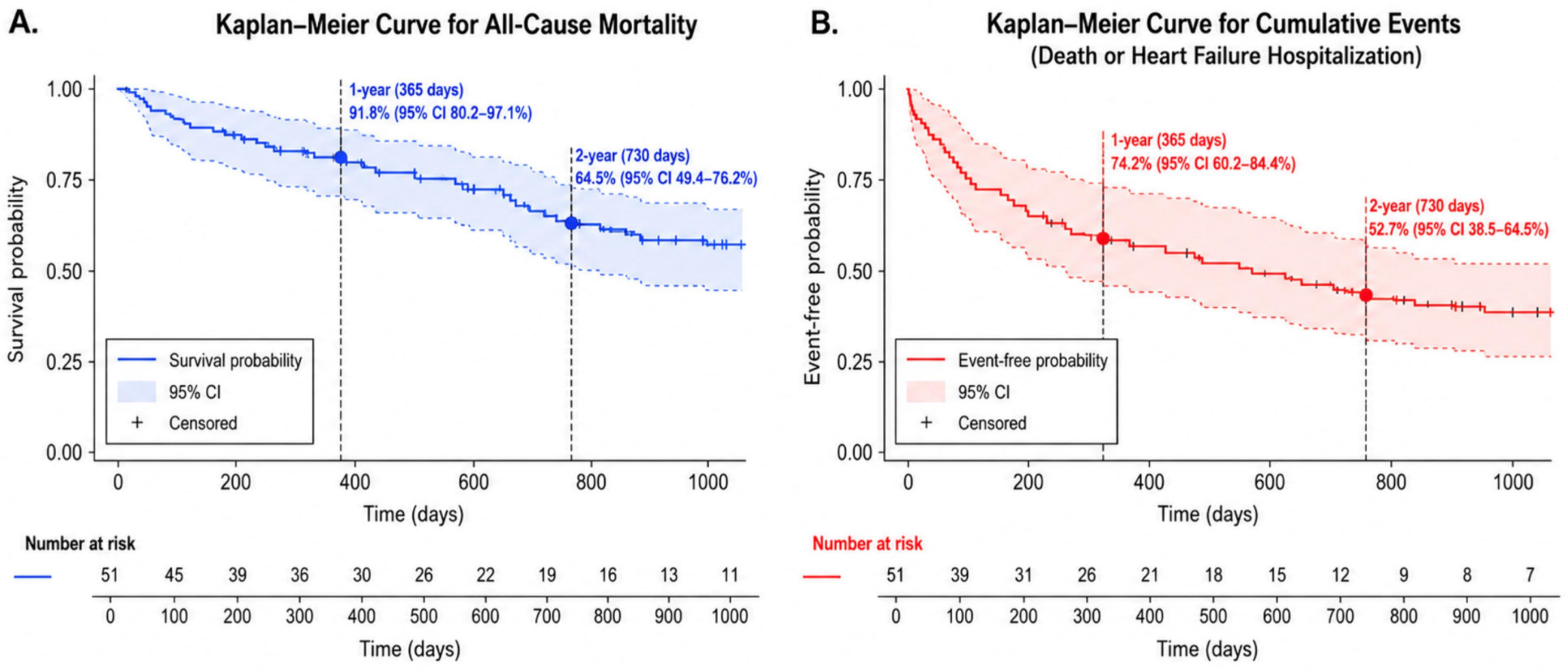
Kaplan–meier curves. **(A)** All-cause mortality. The solid line represents the estimated survival probability over time. The shaded area indicates the 95% confidence interval (CI). Tick marks indicate censored observations. The table below the curve reports the number of patients at risk at selected time points. Estimated survival was 91.8% at 1 year and 64.5% at 2 years. **(B)** Cumulative events, defined as all-cause mortality or heart-failure rehospitalization. The solid line represents the estimated event-free survival probability over time. The shaded area indicates the 95% CI. Tick marks indicate censored observations. The table below the curve reports the number of patients at risk at selected time points. Estimated freedom from cumulative events was 74.2% at 1 year and 52.7% at 2 years.

Median clinical follow-up was 607 days (IQR 289–906). Estimated 1-year and 2-year survival was 91.8% and 64.5%, with 74.2% and 52.7% freedom from mortality or heart failure rehospitalization (Figure 2).

### Logistic regression analysis for mortality and heart failure reshospitalization

Univariate comparison between surviving and deceased patients is presented in Table 1. In the logistic regression mortality model, both TRIVALVE score and baseline hemoglobin were significantly associated with mortality. Higher TRIVALVE scores were associated with increased odds of death, whereas higher baseline hemoglobin levels were associated with reduced odds of death. Model calibration was acceptable according to the Hosmer–Lemeshow goodness-of-fit test (*p* = 0.7) (Table 2). In the TRIVALVE score components-based analysis, atrial fibrillation and signs of RV failure before TR intervention were independently associated with mortality. Model calibration was acceptable according to the Hosmer–Lemeshow goodness-of-fit test (*p* = 0.4) (Table 3).

**Table 1:** Baseline characteristics and univariate comparison– mortality (alive vs. dead).

Variable	Alive (*n* = 39)	Dead (*n* = 15)	*p*-value
*Continuous variables*			
BMI (kg/m^2^)	27.5 ± 5.9	25.3 ± 4.8	0.5
EuroSCORE II (%)	6.2 ± 5.6	7.8 ± 5.5	0.3
TRIVALVE-score	2.0 ± 1.0	2.8 ± 0.8	**0.02**
Hemoglobin (g/dL)	12.7 ± 1.5	11.3 ± 2.3	**0.01**
Creatinine (mg/dL)	1.3 ± 0.6	1.6 ± 0.9	0.1
GOT (U/L)	28.1 ± 11.4	27.1 ± 7.0	0.7
gGT (U/L)	62.6 ± 59.3	79.2 ± 49.3	0.3
sPAP (mmHg)	45.2 ± 12.2	49.8 ± 17.5	0.3
Tapse/sPAP	0.4 ± 0.1	0.4 ± 0.1	0.9
Tapse pre-TC (mm)	18.3 ± 5.7	18.7 ± 4.3	0.8
TR volume (mL)	78.5 ± 28.2	75.7 ± 20.7	0.7
TR vena-contracta (mm)	10.4 ± 3.4	9.3 ± 1.4	0.3
TR-EROA (cm^2^)	0.7 ± 0.3	0.8 ± 0.3	0.7

Categorical variables	Alive	Dead	*p*-value
Male/female sex	11/28	8/7	0.08
AF before TCLIP	17 (43.6%)	13 (86.7%)	**0.004**
GFR <30	7 (17.9%)	3 (20.0%)	1.0
Elevated gGT/bilirubin	14 (35.9%)	9 (60.0%)	0.1
Signs of right HF	18 (46.2%)	12 (80.0%)	**0.02**
LVEF <50%	8 (20.5%)	5 (33.0%)	0.2
Coronary artery disease	13 (33.3%)	8 (53.3%)	0.1
COPD	9 (23.1%)	5 (33.3%)	0.3
Pulmonary hypertension	38 (97.4%)	14 (93.3%)	0.5
pAVK	4 (10.3%)	2 (13.3%)	1.0
Diabetes mellitus	11 (28.2%)	4 (26.7%)	0.6
Hypertension (aHT)	36 (92.3%)	13 (86.7%)	0.4

*p*-values in bold are statistically significant.

**Table 2:** Logistic regression model for overall mortality.

Variable	OR	95% CI	*p*-value
TRIVALVE score	2.19	1.06–4.53	0.034
Baseline hemoglobin	0.64	0.43–0.96	0.029

Model calibration: Hosmer–Lemeshow *p* = 0.786.

**Table 3:** Logistic regression model for overall mortality using individual TRIVALVE components.

Variable	OR	95% CI	*p*-value
AF baseline before TCLIP	9.0	1.64–50.0	0.01
Signs of right heart failure	5.0	1.08–25.0	0.04

Model calibration: Hosmer–Lemeshow *p* = 0.442.

After univariate analysis (Table 4), in the logistic regression to identify determinants for cumulative adverse events, TRIVALVE score remained significantly associated with adverse outcomes, while baseline hemoglobin showed a borderline inverse association. Calibration was acceptable (Hosmer–Lemeshow *p* = 0.739) (Table 5).

**Table 4:** Baseline characteristics and univariate analysis– mortality and/or heart failure rehospitalization .

Variable	No events (*n* = 35)	Events (*n* = 19)	*p*-value
*Continuous variables*			
BMI (kg/m^2^)	28.0 ± 5.9	24.9 ± 4.7	0.06
EuroSCORE II (%)	6.0 ± 5.7	7.8 ± 5.2	0.2
TRIVALVE-score	1.9 ± 1.0	2.7 ± 0.8	**0.03**
Hemoglobin (g/dL)	12.8 ± 1.6	11.6 ± 2.1	**0.02**
Creatinine (mg/dL)	1.3 ± 0.7	1.5 ± 0.8	0.2
GOT (U/L)	27.2 ± 11.2	29.0 ± 8.4	0.5
gGT (U/L)	55.7 ± 53.5	87.2 ± 56.9	0.06
sPAP (mmHg)	48.42 ± 11.8	49.5 ± 17.0	0.3
Tapse/sPAP	0.4 ± 0.1	0.4 ± 0.1	0.9
Tapse pre-TC (mm)	18.6 ± 6.0	18.1 ± 4.1	0.7
TR volume (mL)	78.6 ± 28.7	75.7 ± 19.7	0.7
TR vena-contracta (mm)	10.2 ± 3.3	10.1 ± 2.2	0.9
TR-EROA (cm^2^)	0.7 ± 0.3	0.8 ± 0.3	0.2

Categorical variables	No events	Events	*p*-value
Male/female sex	10/25	9/10	0.1
AF before TCLIP	14 (40.0%)	16 (84.2%)	**0.002**
GFR <30	6 (17.1%)	4 (21.1%)	0.7
Elevated gGT/bilirubin	11 (31.4%)	12 (63.2%)	**0.02**
Signs of right HF	15 (42.9%)	15 (78.9%)	**0.01**
LVEF <50%	7 (20.0%)	6 (31.6%)	0.3
Coronary artery disease	12 (34.3%)	9 (47.4%)	0.3
COPD	9 (25.7%)	5 (26.3%)	0.6
Pulmonary hypertension	34 (97.1%)	18 (94.7%)	0.5
pAVK	4 (11.4%)	2 (10.5%)	0.6
Diabetes mellitus	10 (28.6%)	5 (26.3%)	0.6
Hypertension (aHT)	32 (91.4%)	17 (89.5%)	0.6

*p*-values in bold are statistically significant.

**Table 5:** Logistic regression model for cumulative adverse events.

Variable	OR	95% CI	*p*-value
TRIVALVE score	2.53	1.26–5.09	0.009
Baseline hemoglobin	0.68	0.47–1.00	0.053

Model calibration: Hosmer–Lemeshow *p* = 0.739.

In the exploratory component-based model, selected individual components of the TRIVALVE score were significantly associated with cumulative adverse events. However, the Hosmer–Lemeshow test was significant (*p* = 0.033), indicating less satisfactory calibration. Accordingly, these findings should be interpreted as exploratory and hypothesis-generating (Table 6).

**Table 6:** Logistic regression model for cumulative adverse events using individual TRIVALVE components.

Variable	OR	95% CI	*p*-value
AF baseline before TCLIP	9.09	1.96–33.33	0.004
Signs of right heart failure	5.56	1.35–25.00	0.017

Model calibration: Hosmer–Lemeshow *p* = 0.033.

### Mitral and tricuspid valve function at discharge and follow-up outcomes

**Figure 3:**
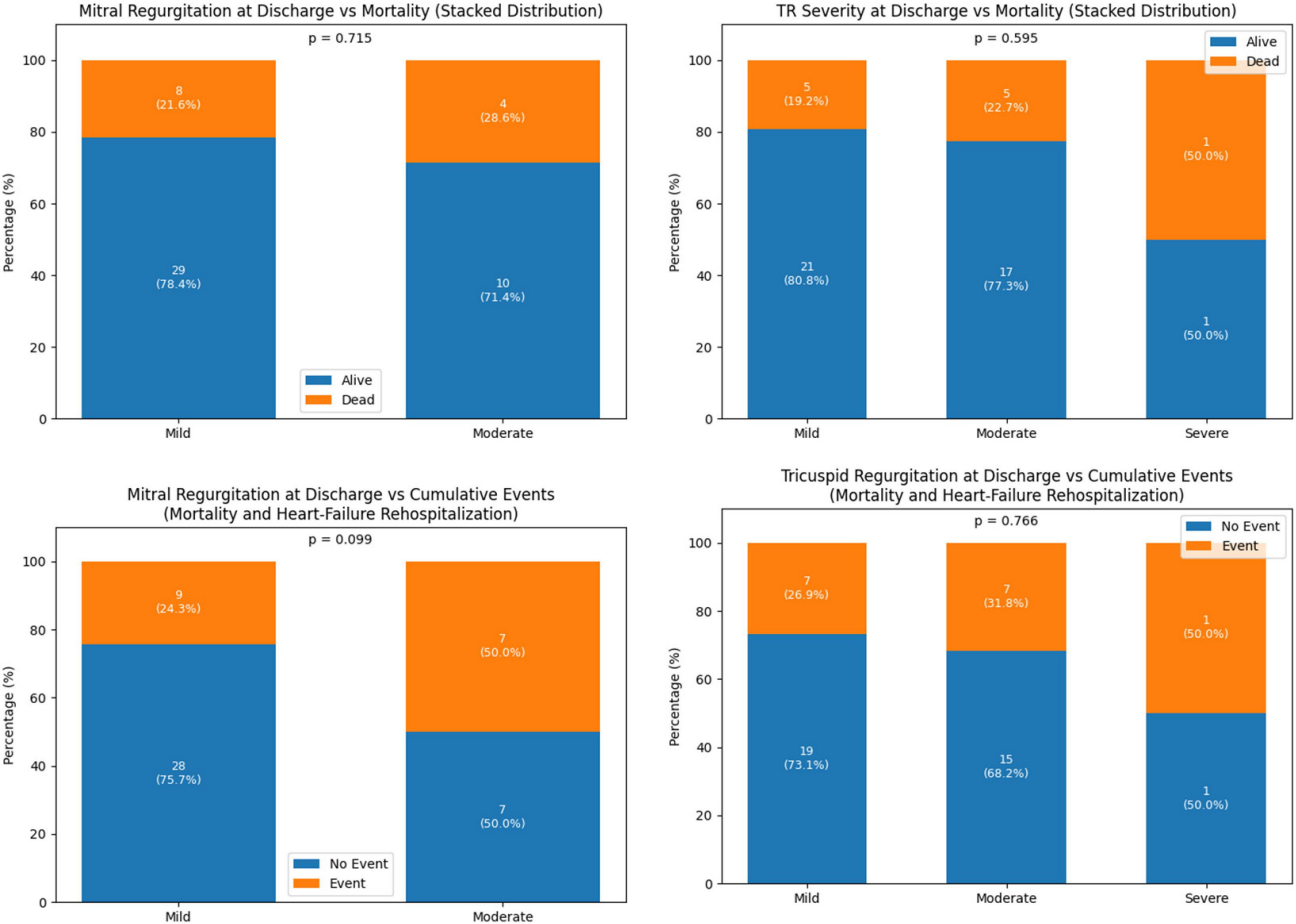
No significant association was observed between MR and TR severity at discharge and follow-up mortality or cumulative events.

Figure 3 summarizes degree of residual MR and TR in discharged patients and follow-up outcomes. No significant association was observed between MR and TR severity at discharge and mortality or cumulative events at follow-up.

### Exploratory predictive modeling for follow-up mortality and cumulative events

For mortality prediction, AUCs were 0.741 (95% CI 0.566–0.916) for logistic regression, 0.799 (95% CI 0.638–0.960) for random forest, and 0.726 (95% CI 0.548–0.904) for gradient boosting.

For cumulative adverse events, AUCs were 0.783 (95% CI 0.635–0.931) for logistic regression, 0.847 (95% CI 0.718–0.976) for random forest, and 0.714 (95% CI 0.553–0.875) for gradient boosting.

**Figure 4:**
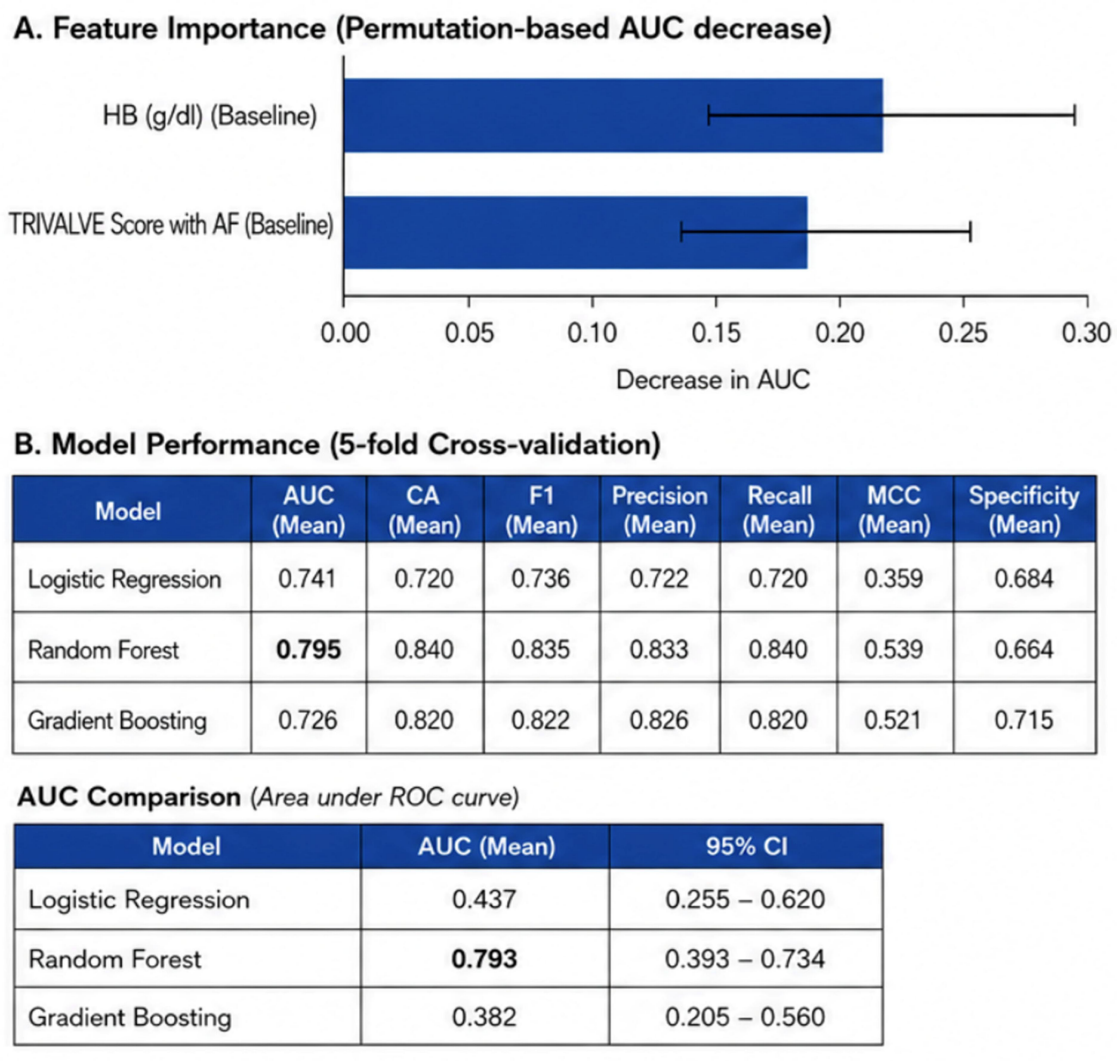
Predictive performance of machine-learning models for follow-up mortality. **(A)** Feature importance assessed by permutation-based decrease in the area under the receiver operating characteristic curve (AUC). Error bars represent variability across the 5-fold cross-validation procedure. **(B)** Predictive performance of logistic regression, random forest, and gradient boosting models evaluated using stratified 5-fold cross-validation. AUC, Area Under the Receiver Operating Characteristic Curve; CA, Classification Accuracy; F1, Harmonic Mean of Precision and Recall (F1-score); MCC, Matthews Correlation Coefficient; CI, Confidence Interval.

**Figure 5:**
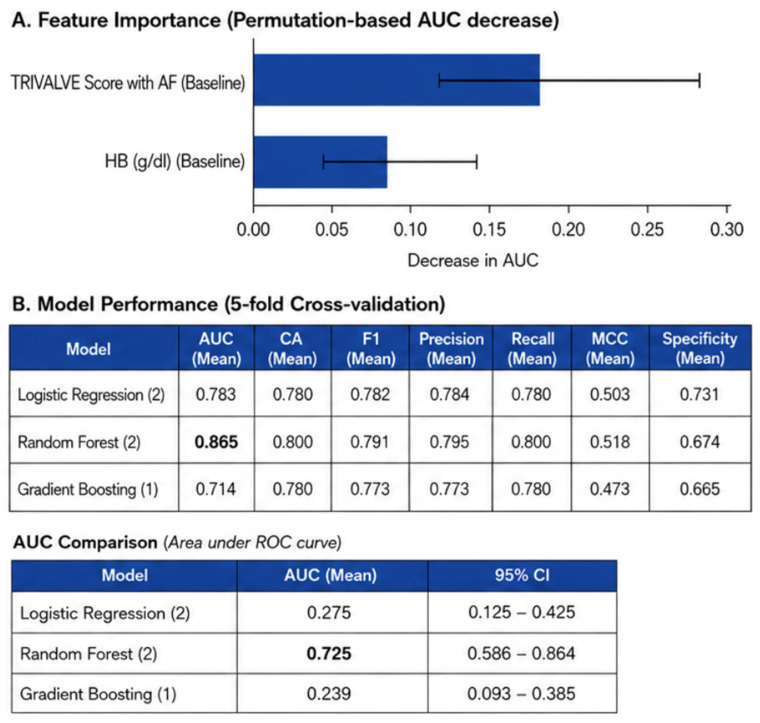
Predictive performance of machine-learning models for cumulative adverse events (all-cause mortality and heart-failure rehospitalization). **(A)** Feature importance assessed by permutation-based decrease in the area under the receiver operating characteristic curve (AUC). Error bars represent variability across the 5-fold cross-validation procedure. **(B)** Predictive performance of logistic regression, random forest, and gradient boosting models evaluated using stratified 5-fold cross-validation. AUC, Area Under the Receiver Operating Characteristic Curve; CA, Classification Accuracy; F1, Harmonic Mean of Precision and Recall (F1-score); MCC, Matthews Correlation Coefficient; CI, Confidence Interval.

Baseline hemoglobin level was the feature with stronger prediction for follow-up mortality (Figure 4), and baseline TRIVALVE score was the feature with stronger prediction for follow-up cumulative events (Figure 5).

## Discussion

The evidence supporting a dual-valve transcatheter strategy still rests largely on observational data (9–12). Our findings add to this field by showing that in patients undergoing combined MitraClip® followed by TriClip®, outcomes are closely linked to the pre-existing burden of comorbidity and frailty, and not simply to the fact that both regurgitant lesions were treated.

A first important message from this study is that staged dual-valve TEER appears to be a clinically meaningful strategy in a very high-risk cohort. This is relevant because patients with combined MR/TR frequently present late, after prolonged exposure to venous congestion, pulmonary hypertension, atrial remodeling, and RV dysfunction. Prior combined-valve observational studies suggested that concomitant mitral-tricuspid TEER may improve symptoms and, in some cohorts, survival or heart-failure-related outcomes compared with isolated MR treatment (9, 10). Our experience extends that concept with longer follow-up and supports the view that treating both valves percutaneously is feasible and clinically justified in carefully selected patients. At the same time, the residual event burden in our cohort underscores that valve correction does not fully reverse the natural history once biventricular and systemic congestion are advanced.

Among the clinical variables baseline hemoglobin was with the TRIVALVE-Score an independent predictor of follow-up mortality. This suggests that long-term survival after combined dual-valve TEER may be influenced not only by valvular and right-heart disease severity, but also by systemic vulnerability. In advanced MR/TR populations, lower hemoglobin likely reflects a broader syndrome of chronic illness, frailty, venous congestion, renal dysfunction, inflammation, or reduced physiological reserve. In other words, death during follow-up may be more closely linked to the patient's overall biological resilience than to valvular disease burden alone.

By contrast, cumulative events, defined as death or heart failure rehospitalization, were more strongly independently predicted by TRIVALVE score. This is clinically plausible. TRIVALVE was specifically developed to predict mortality and heart failure hospitalization after transcatheter tricuspid intervention and incorporates markers of advanced right-heart disease, including atrial fibrillation, right-heart failure, hepatic dysfunction, renal dysfunction, and ventricular impairment (14). Its association with cumulative events in the present study supports the idea that recurrent decompensation after combined TEER is strongly influenced by the burden of right-sided congestion and chronic systemic venous disease rather than by procedural success alone.

The component-level analysis further refines this interpretation. When the total TRIVALVE score was replaced by selected individual components, baseline atrial fibrillation and signs of heart failure at time of TR treatment emerged as the only independent predictors of cumulative events. Although these findings must be interpreted cautiously, as demonstrated by the wide confidence interval and poor calibration of the model, some clinical hypothesis can be drawn.

In particular, the presence of atrial fibrillation may capture a particularly relevant aspect of disease severity in this setting. In combined MR/TR, atrial fibrillation likely reflects chronic atrial remodeling, long-standing volume and pressure overload, and a more advanced congestive phenotype, identifying patients who remain particularly vulnerable to recurrent heart failure episodes despite technically successful dual-valve treatment.

Finally, the added value of the predictive modeling in this study is not to redefine which variables are important, but to show that combining markers of systemic vulnerability (hemoglobin) and right-heart disease burden (TRIVALVE components) allows a reasonable discrimination of patients at risk of adverse outcomes. In other words, standard logistic regression explains “why” events occur, whereas predictive modeling explores “how well” we can identify the patients in whom these events will occur.

Taken together, these results suggest that staged MitraClip®/TriClip® therapy can be clinically meaningful in a very high-risk population, but prognosis remains strongly conditioned by pre-existing disease stage. Mortality appears to be linked more closely to systemic vulnerability, whereas recurrent events seem to be driven more directly by the burden of right-heart disease. This reinforces the concept that patient selection should not rely solely on anatomical suitability for clipping, but rather on an integrated evaluation of systemic condition and right-heart disease severity.

### Limitations

The presented study is a single-center analysis of 54 patients with a modest number of events, so the multivariable associations are hypothesis-generating rather than validated predictors. The cohort combines staged and a small number of simultaneous procedures. Although the exploratory machine-learning models showed moderate discrimination, the 95% confidence intervals were wide, reflecting the small sample size and limited number of events. Moreover, external validation and consequent fine-tuning are missing.

Therefore, the model should not be considered evidence of a clinically deployable prediction tool.

## Conclusion

Despite the constraints of a limited sample size, the study describes a still uncommon and poorly characterized population, with consecutive enrollment, core-laboratory echocardiography, and a full-cohort analysis, and it offers a basis for the larger multicenter studies this field now needs.

In patients undergoing combined MitraClip-/ and TriClip-therapy, follow-up mortality and cumulative clinical events appear to reflect different dimensions of disease severity. Baseline hemoglobin identifies systemic vulnerability associated with mortality, whereas TRIVALVE score and, at component level, atrial fibrillation and right heart failure, identify a right-heart disease phenotype prone to recurrent decompensation. These findings support a more integrated approach to patient selection and risk stratification in combined MR/TR disease.

## Data Availability

The raw data supporting the conclusions of this article will be made available by the authors, without undue reservation.
